# Non-melanoma skin cancer: United Kingdom National Multidisciplinary Guidelines

**DOI:** 10.1017/S0022215116000554

**Published:** 2016-05

**Authors:** C Newlands, R Currie, A Memon, S Whitaker, T Woolford

**Affiliations:** 1 Department of Oral and Maxillofacial Surgery, Royal Surrey County Hospital, Guildford, UK; 2 Department of Oral and Maxillofacial Surgery, Ayrshire and Arran Health Board, UK; 3 Department of Dermatology, Southport and Ormskirk NHS Trust, Ormskirk, UK; 4 Department of Oncology, Royal Surrey County Hospital, Guildford, UK; 5 Department of Otolaryngology-Head and Neck Surgery, Manchester Royal Infirmary, Oxford Road, Manchester, UK

## Abstract

This is the official guideline endorsed by the specialty associations involved in the care of head and neck cancer patients in the UK. This paper provides consensus recommendations on the management of cutaneous basal cell carcinoma and squamous cell carcinoma in the head and neck region on the basis of current evidence.

**Recommendations:**

• Royal College of Pathologists minimum datasets for NMSC should be adhered to in order to improve patient care and help work-force planning in pathology departments. (G)

• Tumour depth is of critical importance in identifying high-risk cutaneous squamous cell carcinoma (cSCC), and should be reported in all cases. (R)

• Appropriate imaging to determine the extent of primary NMSC is indicated when peri-neural involvement or bony invasion is suspected. (R)

• In the clinically N0 neck, radiological imaging is not beneficial, and a policy of watchful waiting and patient education can be adopted. (R)

• Patients with high-risk NMSC should be treated by members of a skin cancer multidisciplinary team (MDT) in secondary care. (G)

• Non-infiltrative basal cell carcinoma (BCC) <2 cm in size should be excised with a margin of 4–5 mm. Smaller margins (2–3 mm) may be taken in sites where reconstructive options are limited, when reconstruction should be delayed. (R)

• Where there is a high risk of recurrence, delayed reconstruction or Mohs micrographic surgery should be used. (R)

• Surgical excision of low-risk cSCC with a margin of 4 mm or greater is the treatment of choice. (R)

• High-risk cSCC should be excised with a margin of 6 mm or greater. (R).

• Mohs micrographic surgery has a role in some high-risk cSCC cases following MDT discussion. (R)

• Delayed reconstruction should be used in high-risk cSCC. (G)

• Intra-operative conventional frozen section in cSCC is not recommended. (G)

• Radiotherapy (RT) is an effective therapy for primary BCC and cSCC. (R)

• Re-excision should be carried out for incompletely excised high-risk BCC or where there is deep margin involvement. (R)

• Incompletely excised high-risk cSCC should be re-excised. (R)

• Further surgery should involve confirmed marginal clearance before reconstruction. (R)

• P+ N0 disease: Resection should include involved parotid tissue, combined with levels I–III neck dissection, to include the external jugular node. (R)

• P+ N+ disease: Resection should include level V if that level is clinically or radiologically involved. (R)

• Adjuvant RT should include level V if not dissected. (R)

• P0 N+ disease: Anterior neck disease should be managed with levels I–IV neck dissection to include the external jugular node. (R)

• P0 N+ posterior echelon nodal disease (i.e. occipital or post-auricular) should undergo dissection of levels II–V, with sparing of level I. (R)

• Consider treatment of the ipsilateral parotid if the primary site is the anterior scalp, temple or forehead. (R)

• All patients should receive education in self-examination and skin cancer prevention measures. (G)

• Patients who have had a single completely excised BCC or low-risk cSCC can be discharged after a single post-operative visit. (G)

• Patients with an excised high-risk cSCC should be reviewed three to six monthly for two years, with further annual review depending upon clinical risk. (G)

• Those with recurrent or multiple BCCs should be offered annual review. (G)

## Introduction

The incidence of all types of skin cancer is increasing. The non-melanoma skin cancers (NMSCs) are mostly basal cell carcinoma (BCC), the commonest human cancer in Caucasians, and cutaneous squamous cell carcinoma (cSCC). Over 80 per cent of these tumours occur on the skin of the head and neck. Most NMSC is easily curable. Death is rare; when it occurs, it does so from metastatic cSCC, or from local invasion by neglected BCC or cSCC. The majority of research regarding the management of skin cancer relates to populations of Caucasians in Australia and North America, and different patterns of disease are likely to exist in Europe and the UK. There are no large prospective randomised, controlled trials  in which different treatments of NMSC have been compared. Organisation of skin cancer services including the treatment of NMSC within the UK National Health Service is determined by National Institute for Health and Care Excellence (NICE) guidance.^1^ This section discusses the management of NMSC, confined to BCC and cSCC of the head and neck. It briefly outlines the management of the primary lesion, and discusses the investigation and treatment of regional metastatic cSCC. Squamous cell carcinoma of the lip is dealt with elsewhere in the guidelines. The reader is advised to access current guidelines referenced in this document for further information on the management of NMSC.^2^^–^^6^

## Epidemiology and aetiology

The incidence of NMCS is underreported in the UK due to inconsistent data collection. The incidence is known to be rising and is estimated to do so until 2040. Non-melanoma skin cancer is more common in men, and with increasing age. The age shift in the population has resulted in an overall increase in total number of skin cancers.

The major predisposing factor for the development of NMSC is chronic sunshine exposure, particularly in childhood. Other common factors include fair skin, other forms of ionising radiation, immunosuppression, previous skin malignancy and premalignant states, such as multiple actinic keratoses.

Immunosuppressed patients with skin cancers comprise mainly transplant patients and those with chronic haematological malignancies. These patients frequently develop multiple skin cancers, which are often aggressive in nature. Skin cancers comprise 40–50 per cent of post-transplant malignancies. There is an increased risk of skin cancer in patients who are taking anti-tumour necrosis factor drugs. Genetic conditions and exposure to sensitising chemicals are rare causes of NMSC as is the occurrence of cSCC in chronic wounds.

## Presentation and diagnosis of NMSC

### Basal cell carcinoma

Nodular lesions are the most common form of BCC. Morphoeic BCCs are found almost exclusively on the head and neck, the commonest single site being the nose. Superficial BCCs are predominantly found on the trunk. Nodular BCC may have clinical cystic or pigmented variants. Basal cell carcinoma has a number of well-described histological subtypes.^7^

**Table I:** The low risk and high risk

Low risk	High risk
1. Nodular	1. Morphoeic/infiltrative
2. Superficial	2. Micronodular
	3. Basosquamous

The 2014 Royal College of Pathology^7^ dataset adopts the term ‘infiltrative BCC’ for all high-risk histological variants and notes that many BCCs contain both high- and low-risk subtypes (Table 1).

### Cutaneous squamous cell carcinoma

Cutaneous squamous cell carcinoma typically presents as an indurated nodular keratinising or crusted tumour that may ulcerate, or it may present as an ulcer without evidence of keratinisation. Cutaneous squamous cell carcinoma of the nasal vestibule or of the ear canal is often diagnosed late, with resulting poor prognosis, as it can be misdiagnosed as other common conditions.

### Diagnosis

Diagnosis of NMSC is usually clinical, with subsequent histological confirmation following excision. The ‘stretch test’ has been shown to improve diagnostic accuracy in BCC. Dermoscopy improves initial diagnostic rate in all NMSC and may be of some assistance in determining a BCC sub-type. Pre-excisional tissue diagnosis can be indicated particularly if a graft or flap will be required for reconstruction, or in an anatomically complex area such as the nose. In most circumstances, this is best achieved by punch, incisional or shave biopsy under local anaesthetic. Shave biopsy is undesirable in possible cutaneous melanoma. Exfoliative cytology has a high diagnostic accuracy in NMSC, particularly where the tumour is ulcerated, and can be of use to guide management where surgical biopsy may be difficult, such as in the very elderly. A tissue diagnosis should also be obtained prior to radiotherapy (RT).

**Recommendation**

• Diagnosis of NMSC is usually clinical. Biopsy (or exfoliative cytology) is recommended where the clinical diagnosis is in doubt, or where histological features may influence treatment, and prior to radiation therapy (G)

## High-risk features of NMSC

Some clinical and histological features are indicative of aggressive tumour behaviour.

### High-risk features of BCC for recurrence:

• Tumour size >2 cm
• Tumour site (the central face)
• Poorly defined clinical margins
• High-risk histological sub-type
• Histological features of aggression; peri-neural or peri-vascular involvement
• Failure of previous treatment (the tumour is a recurrence)
• Immunosuppression.

### High-risk features of cSCC for recurrence and metastasis:

• Size >2 cm
• Failure of previous treatment
• Immunosuppression
• Depth or invasion >2 mm thickness*
• Clark level >4*
• Peri-neural invasion*
• Primary site ear or hair-bearing lip*
• Poorly differentiated or undifferentiated*.

*Determined as high risk in Tumour–Node–Metastasis (TNM) Classification of Malignant Tumours, 7th Edition.

Of note, tumour depth is highly predictive for metastasis and local recurrence. Cutaneous squamous cell carcinoma less than 2 mm in depth has little or no metastatic potential. In cSCC 2.1–6.0 mm thick, the rate of metastasis is 4 per cent and for thickness greater than 6.0 mm the rate is 16 per cent. Tumours invading the sub-cutaneous fat have metastatic rates up to 46 per cent.

NICE^5^ and the Royal College of Pathologists^7^ use greater than 4 mm tumour depth or invasion into subcutaneous fat as indicators for referral to the MDT. The 7th edition of TNM Classification of Malignant Tumours^8^ uses >2 mm tumour depth as a high-risk factor. There is a wide range of malignant behaviour of cSCC; head and neck surgeons are likely to deal with a higher proportion of high-risk tumours.

**Recommendations**

• Royal College of Pathologists minimum datasets for NMSC should be adhered to in order to improve patient care and help work-force planning in pathology departments (G)
• Tumour depth is of critical importance in identifying high-risk cSCC, and should be reported in all cases (R)

**Table II:** T staging for CSCC and other cutaneous carcinomas

TX	Primary tumour cannot be assessed
T0	No evidence of primary tumour
T	Is carcinoma *in situ*?
T1	Tumour 20 mm or less in greatest dimension and (with the exception of BCC*) with less than two high-risk features*
T2	Tumour greater than 20 mm in greatest dimension or (with the exception of BCC*) any size and with two or more high-risk features*
T3	Tumour with invasion of maxilla, mandible, orbit or temporal bone
T4	Tumour with invasion of skeleton (axial or appendicular) or peri-neural invasion of skull base

* Rarely applies to BCC and not accordingly included in staging by The Royal College of Pathologists.

## Staging

The most widely adopted staging system for staging cSCC and BCC is the TNM Classification of Malignant Tumours, 7th Edition (Table II).^8^ Skin cancers of the eyelid, and Merkel cell carcinomas are included elsewhere.

Imaging to determine the extent of primary NMSC may be indicated when peri-neural involvement (magnetic resonance imaging) or bony invasion (computed tomography) is suspected. There is no evidence to support cross-sectional imaging in the clinically node negative patient.

In the clinically node positive patient, further assessment and management is as per the guidelines set out elsewhere in these guidelines, with the following additional points for consideration.

• Cross-sectional imaging should include the parotid.
• Clinically enlarged nodes should be examined initially by fine needle aspiration cytology (FNAC), ideally ultrasound guided. This can be repeated if negative, where clinical suspicion remains.
• Removal of a suspicious node for which FNAC has been non-diagnostic can be carried out via a considered incision which can be incorporated into a future neck dissection approach. This will enable accurate staging of a patient prior to therapeutic neck dissection.
• Sentinel node biopsy for the detection of metastatic disease in high-risk cSCC is only used within clinical trials.^9^

**Recommendations**

• Appropriate imaging to determine the extent of primary NMSC is indicated when peri-neural involvement or bony invasion is suspected (R)
• In the clinically N0 neck, radiological imaging is not beneficial, and a policy of watchful waiting and patient education can be adopted (R)

## The role of the multidisciplinary team

The importance of multidisciplinary working relationships in the management of high-risk NMSC is paramount and patients should be treated by members of a skin cancer MDT. Low-risk BCC is treated in some regions by community practitioners as per updated NICE guidance.^5^ Lesions above the clavicle are specifically excluded from this group, and these patients should receive treatment in secondary care. Cancer networks should establish two levels of MDTs to care for patients, with high-risk cSCC and BCC being discussed either at a local skin MDT or regional specialist skin cancer MDT. It is recognised that local and specialist MDT referral pathways will vary from region to region.

Patients in the following groups should be discussed at the skin cancer MDT as per NICE and Scottish Intercollegiate Guidelines Network Guidance; input from the head and neck cancer MDT will be appropriate in the following groups:

• All patients with high-risk cSCCs, cSCCs and BCCs that may involve the excision margins or are recurrent.
• Patients suitable for Mohs surgery.
• Skin cancers in patients who are immunocompromised or those with genetic predisposition.
• Patients with metastatic SCC or BCC diagnosed at presentation or on follow-up.
• Patients who may benefit from RT.
• Patients who may be eligible for entry into clinical trials.
• Specific challenging management issues, such as cognitive impairment or medical comorbidities.

**Recommendation**

• Patients with high-risk NMSC should be treated by members of a skin cancer MDT in secondary care (G)

## Treatment of the primary lesion

### Surgical excision

#### Basal cell carcinoma

Excision with a predetermined margin is the recommended treatment for the majority of BCCs.^10^ Complete excision rates of 85 per cent with a 3 mm clinical margin have been reported and of 95 per cent with a 4–5 mm margin. The stretch test, dermoscopy, loupe magnification and prior curettage, may improve definition of the tumour margin and reduce incomplete excision rates. The deep margin should include fat, but will be determined by tumour extension – it can be clinically assessed at the time of surgery.

Infiltrative and large BCCs have a higher risk of sub-clinical tumour extension.

In the management of BCCs with a high risk of recurrence, reconstruction should be delayed until histological confirmation of clearance has been confirmed, either by Mohs micrographic surgery (MMS), or until the results of paraffin section are available.

**Recommendations**

• Non-infiltrative BCCs <2 cm in size should be excised with a margin of 4–5 mm. Smaller margins (2–3 mm) may be taken in sites where reconstructive options are limited, when reconstruction should be delayed (R)
• Where there is a high risk of recurrence, delayed reconstruction^11^ or MMS should be used (R)

#### Cutaneous squamous cell carcinoma

Surgical excision with a predetermined clinical margin is the recommended treatment for the majority of cSCC. For clinically well-defined, low-risk tumours, a margin of 4 mm will achieve histological clearance in over 95 per cent of cases. In high-risk cSCC, the evidence on peripheral margins required is limited, but at least 6 mm should be included in the resection. The deep margin on the scalp should include the galea at least; the peri-osteum and outer table should be resected if there is clinical or radiological evidence of involvement. Conventional intra-operative frozen section is less accurate than paraffin section and is no longer recommended. The confirmation of histological clearance can be confirmed by awaiting the results of paraffin section, before reconstruction is undertaken. Both excised BCC and cSCC specimens should be marked for orientation in case further resection is required.

#### Mohs micrographic surgery

Mohs micrographic surgery is a precise technique which combines staged resection with comprehensive histological examination of the surgical margin. It is the treatment of choice in high-risk BCC and not only offers superior tumour control (97 per cent five-year cure rates), but better cosmetic outcomes as tissue removal is minimised. Mohs micrographic surgery is used less often for high-risk SCC due to concerns about the possible presence of in transit metastases and skip lesions, and the more challenging histological margin interpretation (permanent sections are more accurate than frozen sections). Disadvantages of MMS include the length of the procedure (which is carried out under local anaesthetic), the need for special equipment and training and the relatively high cost. The availability of the procedure in the UK is at present limited.

**Recommendations**

• Surgical excision of low-risk cSCC with a margin of 4 mm or greater is the treatment of choice (R)
• High-risk cSCC should be excised with a margin of 6 mm or greater (R)
• Mohs micrographic surgery has a role in some high-risk cSCC cases following MDT discussion (R)
• Delayed reconstruction should be used in high-risk cSCC (G)
• Intra-operative conventional frozen section in cSCC is not recommended (G)

### Destructive techniques

#### Curettage and cautery

This can be used by experienced practitioners for small (<4 mm), well-defined BCC with non-aggressive histology in non-critical sites with a five-year cure rate of up to 97 per cent. Curettage and cautery is used in some centres to treat small (<1 cm) low-risk cSCCs with excellent cure rates, but histological clearance cannot be confirmed. Its use should be confined to experienced practitioners in the technique, employing careful case selection criteria. Curettage and cautery is not indicated in recurrent or high-risk NMSC.

#### Cryosurgery

Cryosurgery is used in low-risk BCC. Disadvantages include scarring, difficulty in assessing recurrence and lack of tissue diagnosis or proof of tumour clearance. Good short-term cure rates have been reported for small histologically confirmed cSCC treated by cryosurgery in experienced hands. Prior biopsy is necessary to establish the diagnosis histologically. For this reason, caution should be exercised in the use of cryotherapy for cSCC although it may be an appropriate technique for selected cases especially in very elderly patients and in specialised centres. Cryosurgery is not appropriate for locally recurrent disease or high-risk tumours.

#### Photodynamic therapy

This therapy is effective in low-risk superficial BCC, but with lower oncologic efficacy than surgery in nodular BCC. It is not recommended for other BCC sub-types or for cSCC.

#### Topical 5 per cent imiquimod

This is an immune response modifier which is licensed for and effective in the treatment of small primary superficial BCC.

#### Vismodegib

This drug is licensed for locally advanced or metastatic BCC not suitable for surgery or RT. This new drug is an antagonist for the smoothened G-protein-coupled receptor molecule, and thus inhibits the aberrant signalling pathway involving Hedgehog (Hh) genes. Early trials show efficacy in 50 per cent of BCCs with mean duration of response around nine months. It is a suitable treatment in recurrent, inoperable BCCs post-RT or in patients with Gorlin's syndrome, and in the very rare occurrence of metastatic BCC.

### Radiotherapy in primary NMSC

Radiotherapy is an alternative to surgery for primary BCC and cSCC of the head and neck region in the following scenarios:

• Elderly or frail patients
• Anatomical sites where RT is likely to lead to a superior cosmetic or functional outcome
• Surgery is contraindicated
• Patient choice.

At most head and neck sites, cosmetic outcomes and cure rates with RT are inferior to excisional surgery.

Radiotherapy is normally not used in the following circumstances:

• Patient age over 50 years, due to the risk of second malignancies and inferior cosmetic outcome
• Sites of previous RT
• Cartilage or bone involvement due to risk of radionecrosis
• Over the lateral half of the upper eyelid due to risk of lacrimal gland damage.

Basal cell carcinoma and cSCC are usually treated with low-energy (KV) X-rays, but may be treated with electrons. Alternatively, high-energy (MV) X-rays may be used in the presence of deep extension or tumour fixation. Common fractionation schedules range from five fractions in one week for lesions greater than 2–3 cm; to 9–10 fractions in two to three weeks for intermediate size; and 20–30 fractions over four to six weeks for very large (>6 cm) lesions or where regional lymph node irradiation is also required. The dose is usually higher and a larger margin included in the treatment field when treating cSCC than BCC.^12^^,^^13^

**Recommendation**

• Radiotherapy is an effective treatment for primary BCC and cSCC (R)

### Incomplete margins of excision

Incomplete excision of BCC can occur in the setting of high-risk tumour factors, low operator expertise and when multiple tumours are removed at the same procedure. Incompletely excised NMSC should be discussed at the MDT, as should those with a margin of excision less than 1 mm. Options for management include observation (many low-risk tumours will not recur), re-excision (by standard surgery or with marginal control) and adjuvant treatment (radiation therapy or topical therapy)

British Association of Dermatology recommendations for consideration of re-excision of transected BCC include:

• Anatomically critical site
• Infiltrative histology
• Deep margin involvement
• Flap or graft reconstruction.

Incompletely excised high-risk cSCC should be re-excised to reduce the risk of recurrence and metastasis. In closely excised high-risk cSCC, re-excision or the use of adjuvant RT should be discussed at the MDT and may be influenced by local anatomy, and reconstructive factors. Where further treatment of NMSC is indicated and re-excision is not possible, adjuvant RT is indicated to decrease recurrence rates.^14^^,^^15^

If a margin is involved by superficial BCC only, topical imiquimod may be indicated.

**Recommendations**

• Re-excision should be carried out for incompletely excised high-risk BCC or where there is deep margin involvement (R)
• Incompletely excised high-risk cSCC should be re-excised (R)
• Further surgery should involve confirmed marginal clearance before reconstruction (R)

## Management of regional metastatic CSCC

### Patterns of metastasis

The overall regional metastatic rate of cSCC in a UK population has been reported at around 5 per cent.^16^ These rates can be higher in the presence of adverse histological features; for instance, 33 and 47 per cent for poor differentiation or peri-neural infiltration, respectively. Tumour thickness is strongly correlated with risk of nodal metastasis. The presence of metastatic nodal disease is associated with a five-year survival of 35 per cent.

**Fig. 1:**
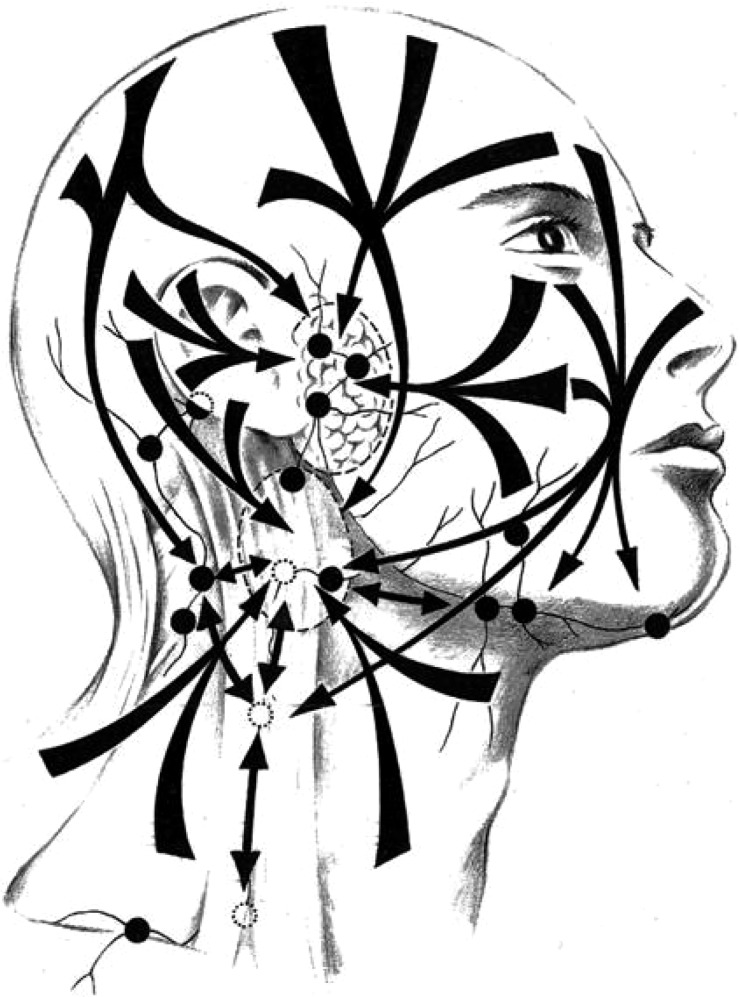
Patterns of metastasis of cSCC to the external jugular node and the superficial lymphatics.

Lymph node metastases of NMSC of the head and neck are known to follow different pathways to the classically understood patterns of mucosal malignancies of the upper aerodigestive tract (Figure 1). The parotid nodes and the superficial lymphatic system need to be addressed, in contrast to mucosal head and neck mucosal malignancies. Sentinel node biopsy studies have shown a high lack of concordance between the primary skin site and the first echelon node. The external jugular node is of particular relevance as it is not included in standard neck dissections for head and neck squamous cell carcinoma.

Over 50 per cent of cSCC occurs on the anterior scalp and forehead and the ears, and the parotid is the site for up to 70 per cent of metastasing cSCC. Where the parotid is involved (P+), there is an increased chance of the neck containing occult and overt metastases (10–35 per cent). In the P+N+ scenario, the incidence of metastases in level V approaches 30 per cent.

N+ P0 disease is seen where the primary site was the face or upper neck or posterior scalp. The posterior scalp is the site for 5 per cent of cSCC, and tumours here will metastasise initially commonly to post-auricular, occipital or level V nodes.^17^^,^^18^ Resection of structures such as the facial nerve, the internal jugular vein, the accessory nerve and the sternocleidomastoid muscle are required in a nodal dissection in the presence of invasion by the malignant process.

### Management of nodal involvement

Surgery is the primary mode of treatment for established nodal involvement and adjuvant RT may improve survival in high-risk cases. The dissection employed should include established nodal involvement and extend to those levels where there is a high risk of occult disease. In most cases, parotid surgery will involve a superficial parotidectomy; deep lobe or facial nerve involvement will require more extensive resection.

**Recommendations**

• P+ N0 disease:
  Resection should include involved parotid tissue, combined with levels I–III neck dissection, to include the external jugular node (R)
• P+ N+ disease:
  Resection should include level V if that level is clinically or radiologically involved (R)
  Adjuvant RT should include level V if not dissected (R)
• P0 N+ disease:
  Anterior neck disease should be managed with a levels I–IV neck dissection to include the external jugular node (R)
  P0 N+ Posterior echelon nodal disease (i.e. occipital or post-auricular) should undergo dissection of levels II–V, with sparing of level I (R)
  Consider treatment of the ipsilateral parotid, if the primary site is the anterior scalp, temple or forehead (R)

### Role of RT in P+ and/or N+ disease

Retrospective studies suggest that locoregional control and survival are improved by adjuvant RT in cases of cSCC where neck involvement is staged greater than N1, or where there is extracapsular spread. Of note, ECS is seen in up to 70 per cent of head and neck cSCC nodal dissection, and therefore consideration can be given to more selectivity in nodal dissection, as post-operative RT will be indicated for the majority of patients.^19^

## Follow-up

Follow-up in secondary care may detect local recurrence, regional metastasis and new skin cancers at an earlier stage. Of note, the risk of a second BCC is 44 per cent, and up to 50 per cent of Australian cSCC patients develop a second cSCC within two years. Minimisation of immunosuppression in an organ transplant patient with multiple or recurrent high-risk cSCC should be considered by the MDT in conjunction with the patient's relevant physician. Oral retinoids can be used for secondary prevention skin cancers in the immunosuppressed.

**Recommendations**

• All patients should receive education in self-examination and skin cancer prevention measures (G)
• Patients who have had a single completely excised BCC or low-risk cSCC can be discharged after a single post-operative visit (G)
• Patients with an excised high-risk cSCC should be reviewed three to six monthly for two years, with further annual review depending upon clinical risk (G)
• Those with recurrent or multiple BCCs should be offered annual review (G)

### Key points

• Diagnosis of NMSC is usually clinical.
• Excisional surgery with predetermined margins is the treatment of choice for the majority of cases.
• Imaging is recommended in large primary tumours, but does not have a role where the regional nodes are clinically N0.
• Reconstruction should be delayed in high risk NMSC.

## References

[1] National Institute for Health and Care Excellence. Improving Outcomes for People with Skin Tumours Including Melanoma. London: National Institute for Health and Care Excellence, 2006 https://www.nice.org.uk/guidance/csg8 (accessed 27 April 2016)

[2] Telfer NR, Colver GB, Morton CA. Guidelines for the management of basal cell carcinoma. Br J Dermatol 2008;159:35–48. http://www.bad.org.uk/library-media%5Cdocuments%5CBCC_2008.pdf (accessed 15 March 2015) 18593385 10.1111/j.1365-2133.2008.08666.x

[3] Management of the Patient with Primary Squamous Cell Carcinoma. London: British Association of Dermatologists, 2009 http://www.bad.org.uk/library-media%5Cdocuments%5CSCC_2009.pdf (accessed 15 March 2015)

[4] Scottish Intercollegiate Guidelines Network (SIGN). Management of Primary Cutaneous Squamous Cell Carcinoma. Edinburgh: SIGN, 2014 (SIGN publication no. 140). http://www.sign.ac.uk/guidelines/fulltext/140/index.html (accessed 15 March 2015)

[5] National Institute for Health and Care Excellence. Improving Outcomes for People with Skin Tumours including Melanoma (update). The Management of Low-risk Basal Cell Carcinomas in the Community. London: National Institute for Health and Care Excellence, 2006 http://www.nice.org.uk/guidance/csgstim/documents/skin-cancer-update-management-of-lowrisk-basal-cell-carcinomas-in-the-community2 (accessed 15 March 2015)

[6] Karia PS, Jambusaria-Pahlajani A, Harrington DP, Murphy GF, Qureshi AA, Schmults CD. Evaluation of American Joint Committee on Cancer, International Union against cancer, and Brigham and Women's Hospital Tumor Staging for Cutaneous Squamous Cell Carcinoma. J Clin Oncol. 2014;32:327–34 24366933 10.1200/JCO.2012.48.5326 PMC3897257

[7] Slater D, Walsh M. Dataset for the Histological Reporting of Primary Invasive Cutaneous Squamous Cell Carcinoma and Regional Lymph Nodes. London: The Royal College of Pathologists, 2014 https://www.rcpath.org/resourceLibrary/g124_datasetsquamous_may14-pdf.html (accessed 27 April 2016)

[8] Edge SB, Byrd DR, Compton CC, Fritz AG, Greene FL, Trotti A, eds. AJCC Cancer Staging Manual, 7th edn. New York: Springer-Verlag, 2009

[9] Rastrelli M, Soteldo J, Zonta M, Trifiro G, Mazzarol G, Vitali GC, Sentinel node biopsy for high-risk cutaneous nonanogenital squamous cell carcinoma: a preliminary result. Eur Surg Res 2010;44:204–8 20523053 10.1159/000312649

[10] Bath-Hextall FJ, Perkins W, Bong J, Williams HC. Interventions for basal cell carcinoma of the skin. Cochrane Database Syst Rev 2007;CD003412 17253489 10.1002/14651858.CD003412.pub2

[11] Niederhagen B, von Lindern JJ, Berge S, Bergé S, Appel T, Reich RH Staged operations for basal cell carcinoma of the face. Br J Oral Maxillofac Surg 2000;38:477–9 11010777 10.1054/bjom.2000.0322

[12] Zagrodnik B, Kempf W, Seifert B, Müller B, Burg G, Urosevic M Superficial radiotherapy for patients with basal cell carcinoma; recurrence rates, histologic subtypes, and expression of p53 andBcl-2. Cancer 2003;98:2708–14 14669293 10.1002/cncr.11798

[13] Veness MJ, Goedjen B, Jambusaria A. Perioperative management of high risk primary cutaneous squamous cell carcinoma: role of radiologic imaging, elective lymph node dissection, sentinel lymph node biopsy, and adjuvant radiotherapy. Curr Derm Rep 2013;2:77–83

[14] Veness MJ, Morgan GJ, Palme CE, Gebski V. Surgery and adjuvant radiotherapy in patients with cutaneous head and neck squamous carcinoma metastatic to lymph nodes; combined treatment should be considered best practice. Laryngoscope 2005;115:870–5 15867656 10.1097/01.MLG.0000158349.64337.ED

[15] Mourouzis C, Boynton A, Grant J, Umar T, Wilson A, McPherson D Cutaneous head and neck SCCs and risk of nodal metastases – UK experience. J Craniomaxillofac Surg 2009;37:443–7 19713116 10.1016/j.jcms.2009.07.007

[16] Schmults CD, Karia PS, Carter JB, Han J, Qureshi AA. Factors predictive of recurrence and death from cutaneous squamous cell carcinoma: a 10-year, single-institution cohort study. JAMA Dermatol 2013;149:541–7 23677079 10.1001/jamadermatol.2013.2139

[17] Vauterin TJ, Veness MJ, Morgan GJ, Poulson MG, O'Brien CJ. Patterns of lymph node spread of cutaneous squamous cell carcinoma of the head and neck. Head Neck 2006;28:785–91 16783833 10.1002/hed.20417

[18] Gurney B, Newlands C. Management of regional metastatic disease in head and neck cutaneous malignancy. 1. Cutaneous squamous cell carcinoma. Br J Oral Maxillofac Surg 2014;52:294–300 24559975 10.1016/j.bjoms.2014.01.015

[19] Mendenhall WM, Amdur RJ, Hinerman RW, Cognetta AB, Mendenhall NP. Radiotherapy for cutaneous squamous and basal cell carcinomas of the head and neck. Laryngoscope 2009;119:1994–9 19688856 10.1002/lary.20608

